# A Participatory Science Approach to Evaluating Factors Associated with the Occurrence of Metals and PFAS in Guatemala City Tap Water

**DOI:** 10.3390/ijerph19106004

**Published:** 2022-05-15

**Authors:** Jennifer Hoponick Redmon, Riley E. Mulhern, Edwin Castellanos, Erica Wood, Andrea McWilliams, Isabel Herrera, Chamindu Liyanapatirana, Frank Weber, Keith Levine, Evan Thorp, Nichole Bynum, Kelly Amato, Maria Andrea Najera Acevedo, Justin Baker, George Van Houtven, Candise Henry, Christopher Wade, AJ Kondash

**Affiliations:** 1 RTI International, Research Triangle Park, NC 27709, USA; ericawood@rti.org (E.W.); acm@rti.org (A.M.); chamindu@rti.org (C.L.); fxw@rti.org (F.W.); levine@rti.org (K.L.); ethorp@rti.org (E.T.); nbynum@rti.org (N.B.); kea@rti.org (K.A.); jsbaker4@ncsu.edu (J.B.); gvh@rti.org (G.V.H.); clhenry@rti.org (C.H.); chwade@rti.org (C.W.); akondash@rti.org (A.K.); 2 Observatorio Económico Sostenible, Universidad del Valle de Guatemala, Guatemala City, Guatemala; ecastell@uvg.edu.gt (E.C.); eblisach@gmail.com (I.H.); manajera@uvg.edu.gt (M.A.N.A.)

**Keywords:** drinking water quality, climate change, sustainability, arsenic, lead, PFAS, Guatemala, participatory science, community-engaged research

## Abstract

Limited information is available regarding chemical water quality at the tap in Guatemala City, preventing individuals, water utilities, and public health authorities from making data-driven decisions related to water quality. To address this need, 113 participants among households served by a range of water providers across the Guatemala City metropolitan area were recruited as participatory scientists to collect first-draw and flushed tap water samples at their residence. Samples were transported to the U.S. and analyzed for 20 metals and 25 per- and polyfluoroalkyl substances (PFAS). At least one metal exceeded the Guatemalan Maximum Permissible Limit (MPL) for drinking water in 63% of households (*n* = 71). Arsenic and lead exceeded the MPL in 33.6% (*n* = 38) and 8.9% (*n* = 10) of samples, respectively. Arsenic was strongly associated with groundwater while lead occurrence was not associated with location, water source, or provider. One or more PFAS were detected in 19% of samples (*n* = 21, range 2.1–64.2 ppt). PFAS were significantly associated with the use of plastic water storage tanks but not with location, water source, or provider. Overall, the high prevalence of arsenic above the MPL in Guatemala City tap water represents a potential health risk that current water treatment processes are not optimized to remove. Furthermore, potential contaminants from premise plumbing and storage, including lead and PFAS, represent additional risks requiring further investigation and public engagement.

## 1. Introduction

**Figure 1:**
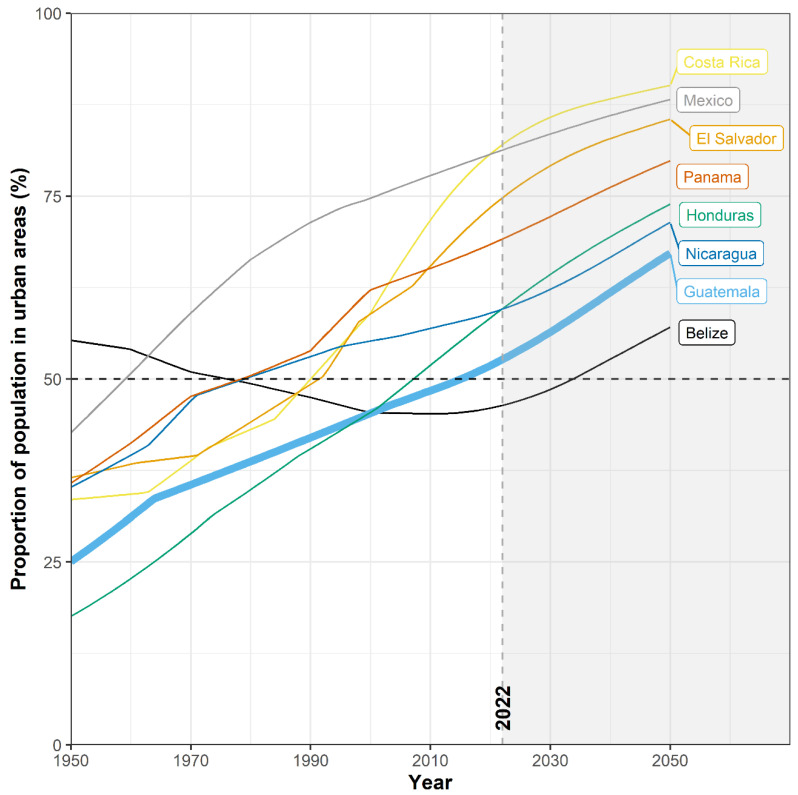
Rates of urbanization in Central American countries 1950 to present, with projected rural/urban proportions shown through 2050. Thick line emphasizes Guatemala trend. Data from World Urbanization Prospects, by the United Nations (UN), Department of Economic and Social Affairs, Population Division, © 2018 UN. Data used with the permission of the UN [6].

Lack of access to food, energy, and water (FEW) in the face of a changing climate could hasten large-scale migration from rural to urban settings worldwide [1]. In Latin America alone, over 10 million people may migrate within their own countries by 2050, away from areas of water insecurity and diminishing crop productivity to urban centers [1]. These pressures are acutely felt in Guatemala and Central America. Uncertainty regarding the rainy season exacerbates challenges for small farmers which, along with meager economic opportunities and limited basic services, has led to mass migration from rural to urban settings [2,3,4,5]. In 2018, the urban population in Guatemala exceeded the rural population for the first time (Figure 1), with approximately 3 million residents in the metropolitan area of Guatemala City, making it the largest city in Central America [6].

Although economies of scale in urban areas allow governments to provide essential FEW services to a larger population at a lower cost than in dispersed, rural areas [7,8], rapid and unplanned urban growth can also affect the quality and efficiency of these same services. For example, increases in unregulated extraction of groundwater resources by private companies, housing developments, and water utilities has caused rapid declines in water table levels below Guatemala City since the 1990s [9]. Flooding from surface runoff in urban zones has also caused millions of dollars in damage to critical water infrastructure in the past two decades [9]. Furthermore, urban runoff and poor or non-existent wastewater management represent drinking water contamination risks [9,10,11]. 

As a result of these coalescing pressures on water infrastructure and resources in urban areas, utilities often struggle to consistently supply quality drinking water and the public generally assumes that tap water is unsafe to drink [12,13,14]. Indeed, only 65% of urban households in Guatemala are estimated to have access to a safely managed drinking water source, and very little water quality data exists for those who have access [15]. A systematic review of studies addressing the FEW nexus in Guatemala reported that drinking water was one of the most frequent concerns [16]. Most of the research on drinking water quality in Guatemala to date, however, has focused on microbial risks in rural areas [17,18,19,20,21,22,23,24]. As the country’s population becomes increasingly urbanized, improved water quality monitoring in urban zones is paramount for achieving Sustainable Development Goal 6 of universal access to safe drinking water. Specifically, water quality monitoring enables local decision-makers to engage the public, improve drinking water quality governance, prioritize infrastructure investments, and develop policies that deliver sustainable FEW services and protect public health [25,26,27]. 

In response to these needs, this study sought to characterize chemical tap water quality in the Guatemala City metropolitan area and identify factors associated with the occurrence of certain chemicals of concern. There are limited data on the occurrence and distribution of metals (including lead) and emerging contaminants such as per- and polyfluoroalkyl substances (PFAS) in urban Guatemalan tap water and in international water, sanitation, and hygiene (WASH) research overall [28]. To our knowledge, only one other peer-reviewed study has reported metals concentrations other than arsenic in Guatemalan tap water [29] and none have evaluated metals other than arsenic in Guatemala City. Additionally, our study represents the first analysis of PFAS in Central American tap water, thus addressing a critical data shortage for these chemicals in Latin America [30,31]. Our participatory science approach allowed for rapid sampling across the city during the COVID-19 pandemic and engaged the community around water quality [32,33]. The results are intended to support decision-making by public health authorities and water service providers in Guatemala and other urban areas throughout Latin America, while also empowering individual residents to understand their water quality and make simple improvements at the tap. 

## 2. Materials and Methods

### 2.1. Recruitment and Questionnaire 

**Figure 2:**
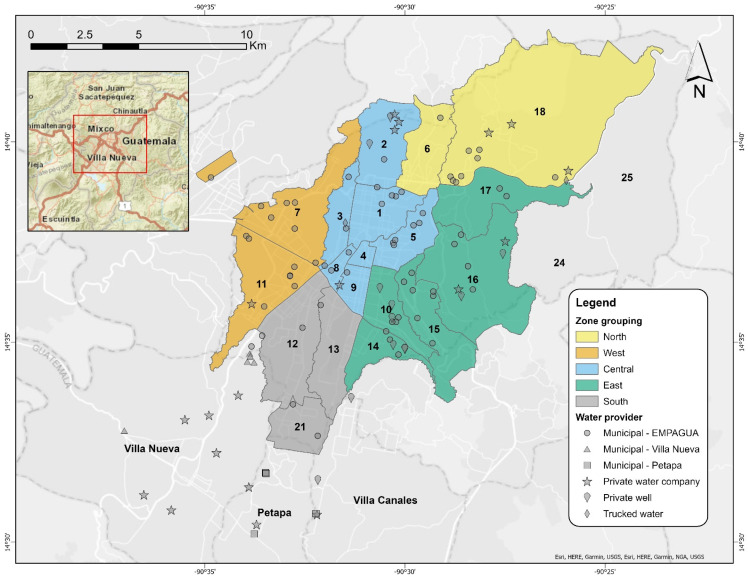
Map of the Guatemala City metropolitan area and the approximate locations and water providers of the study participants. Colors show the different zone groupings. Symbols indicate the water provider.

Potential participants were initially recruited from faculty, staff, and students at the Universidad del Valle de Guatemala (UVG) using an online flyer and survey sent to UVG email addresses, along with hard copies of flyers on campus. A total of 207 potential participants expressed interest. Participants were then selected using a stratified sampling approach to ensure adequate participation across the study area. The Guatemala City metropolitan area consists of 22 distinct zones plus adjoining areas in the municipalities of Villa Nueva, San Miguel Petapa, and Villa Canales to the south. In total, 113 participants submitted water samples, representing 20 of the 22 formal city zones (no UVG-affiliated households were found in zones 24 or 25), Villa Nueva, San Miguel Petapa, and Villa Canales (Figure 2). An online questionnaire was administered to most participants to collect information on socioeconomic status, home ownership, building age, water provider, frequency of water shutoffs, household water storage, and perceptions of water quality (perceptions data forthcoming), with in person completion available for select maintenance staff (see Supplementary Materials (SM) for more details). 

### 2.2. Classification of Water Source

Households served by private wells or private water companies were categorized as being supplied by groundwater. Private water companies around Guatemala City obtain water through one or more privately operated wells and distribute it to a subdivision or condominium and charge the recipients monthly rates. Households served by municipal water providers may be served by groundwater, surface water, or mixed source water, depending on the zone of the city and time of year. Information regarding the water source for households served by the main Guatemala City water utility, Empresa Municipal de Agua (EMPAGUA), was obtained from EMPAGUA engineers according to the water source supplied to the zone of each household during the sampling dates. Information regarding the water source for households served by municipal providers in Villa Nueva, San Miguel Petapa, and Villa Canales was obtained directly from the water utilities. 

### 2.3. Sampling and Analysis

Using provided sampling kits picked up at UVG or sent via courier, participants were trained to collect samples with an online video and written instructions. Participants collected a first-draw sample for metals analysis after at least 8 h of stagnation time in 250 mL HDPE bottles. First-draw samples were collected for metals analysis to evaluate risk from corrosion byproducts in tap water, such as lead, following the requirements for compliance sampling in the U.S. under the Lead and Copper Rule [34]. Due to the possibility of wide variability in lead levels from different sampling approaches and flushing times, first-draw sampling provided a standardized approach for evaluating metals risk in tap water [35]. Participants subsequently ran the water for 3–5 min and collected a PFAS sample and field blank sample in 250 mL polypropylene bottles. Samples were returned to UVG between 21 and 29 March 2021 and refrigerated before return shipment in FedEx cooling boxes to RTI International in Research Triangle Park, North Carolina, United States (U.S.). Samples were received at RTI on 1 April 2021 and analyzed for a panel of 20 metals (Table S1) and 25 PFAS (Table S2) according to methods approved by the U.S. Environmental Protection Agency (EPA). The fully specified conditions for both methods have been described in detail previously [36,37]. 

Briefly, metals were analyzed according to EPA Method 200.8. Turbidity was measured prior to acidification. Per EPA 200.8, if turbidity was less than 1 nephelometric turbidity units (NTU), direct analysis was performed. If turbidity was greater than 1 NTU, the sample was sent through a digestion process. Once turbidity had been determined, the samples were acidified with nitric acid and mixed well. Samples were then held for a minimum of 16 h between acidification and analysis prior to the pH being verified < 2. The samples were analyzed by inductively coupled plasma–mass spectrometry (ICP-MS) using a Thermo Scientific Q instrument equipped with a DX4 autosampler. The samples were stored at ambient temperature pre- and post-analysis. 

PFAS were analyzed according to a modified EPA Method 533. Samples were extracted using a weak anion exchange, polymeric sorbent (Phenomenex #8B-S038-HCH) and then reconstituted in 1 mL of 80% methanol/reagent water (*v*/*v*). Isotope performance standards were added to the extracts and a 10 µL aliquot was analyzed by liquid chromatography/tandem mass spectrometry (LC-MS/MS) using an Agilent 1290 LC system and Agilent 6470 triple quadrupole MS/MS instrument. During the analysis, four PFAS sample bottles were broken, and these samples could not be reported. All PFAS samples were stored under refrigeration prior to extraction.

### 2.4. Statistical Methods

Metals concentrations were compared to Guatemalan drinking water limits [38]. The Guatemalan standards include Maximum Acceptable Limits (MALs) for aesthetic water quality and Maximum Permissible Limits (MPLs) for health-related contaminants. For PFAS, no standards currently exist for drinking water quality in Guatemala. Thus, PFAS levels were compared to the U.S. EPA’s Health Advisory Level (HAL) of 70 parts per trillion (ppt) for PFOA and PFOS, the European Union (EU) Drinking Water Directive limit of 500 ppt for the sum of all PFAS [39], and the more stringent limit set by the International Bottled Water Association (IBWA) of 10 ppt for the sum of PFAS [40]. 

To assess statistical differences in water quality in different parts of the city, the city zones were aggregated into northern, southern, eastern, western, and central categories. Water samples from Villa Nueva, San Miguel Petapa, and Villa Canales were grouped separately. Multiple logistic regression analysis was used to test the significance of various socioeconomic and infrastructure-related variables (Table 1) in predicting the odds of metals exceedances. A separate multiple logistic regression model was developed to evaluate the odds of any PFAS occurring above the reporting limit. Initially, all potential predictors were included in each model and insignificant variables were removed in a stepwise fashion to minimize overfitting. All statistical analyses were conducted in the software R (version 4.1.1). 

## 3. Results

### 3.1. Household Characteristics

Most participants (70%, *n =* 79) obtained their water from municipal providers (Table 1). Of these, 89% received water from the Guatemala City water utility EMPAGUA (*n =* 70). Other households were supplied by private wells serving single households (11%, *n =* 12), private water companies throughout the city (18%, *n =* 20), or had water trucked in (2%, *n* = 2). Most participants were supplied by groundwater (62%, *n =* 70) while 32% were supplied by surface water, 4% by mixed sources and 2% were unknown. A range of home ages was reported, from 2 to over 100 years old (mean = 32 years). Monthly household incomes ranged from less than 4000 Q (approximately 500 USD) to over 24,000 Q (over 3000 USD). Most participants had received some university education or a higher degree (86%, *n =* 97). 

### 3.2. Occurrence of Metals in Tap Water

**Figure 3:**
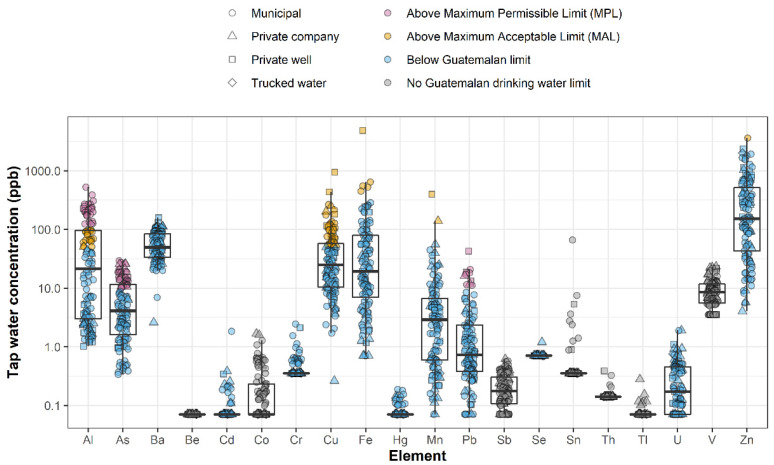
Distribution of metals concentrations in first draw tap water samples among all water providers sampled in the Guatemala City metropolitan area. Horizontal lines of each box indicate the first quartile, median, and third quartile for each metal. Vertical black lines indicate the spread of the data up to ±1.5 times the interquartile range. Symbol colors separate samples by established limits while shapes sort the samples by provider type.

Three metals exceeded Guatemalan health standards (MPLs): aluminum (Al), arsenic (As), and lead (Pb) (Figure 3, Table S3). Overall, tap water in 63% of households (*n* = 71) exceeded the Guatemalan MPL for at least one metal. As and Pb exceeded the MPL (10 parts per billion (ppb) for both metals) in 33.6% (*n* = 38) and 8.9% (*n* = 10) of samples, respectively. As was detected above, the reporting limit in 100% of samples and ranged 0.3–29.3 ppb (mean = 7.4 ppb). Pb was detected above the reporting limit in 94% of samples and ranged <0.1–42.6 ppb (mean = 2.9 ppb). Although Pb exceeded the Guatemalan MPL in only 8% of samples, 42% of samples (*n* = 48) exceeded the American Academy of Pediatrics’ reference level of 1 ppb of Pb in drinking water [42]. Al, although not often regulated in drinking water for health concerns [43], is regulated at a health-permissible level of 100 ppb in Guatemala. Overall, 24% of samples (*n* = 27) exceeded this limit with concentrations ranging 1.0–528 ppb (mean = 70.8 ppb). Five metals also exceeded the Guatemalan aesthetic standard (MAL): Al, copper (Cu), iron (Fe), manganese (Mn), and zinc (Zn). At least one of these metals exceeded the MAL in 64% of households (*n* = 72). 

**Figure 4:**
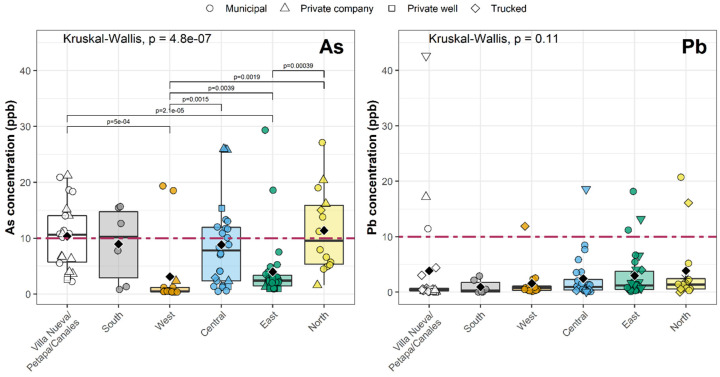
Distribution of As and Pb concentrations in first-draw tap water throughout the Guatemala City metropolitan area. Horizontal lines of each box indicate the first quartile, median, and third quartile for each metal; vertical lines indicate the spread of the data up to ± 1.5 times the interquartile range. Black diamonds show the mean value. Dashed red lines indicate the MPL for As and Pb. Pairwise comparisons highlight significant differences (adjusted for multiple comparisons) between geographic areas using Wilcoxon tests. As showed significant differences between different parts of the city, but Pb did not. Kruskal–Wallis tests evaluate the overall significance of the geographic area on As and Pb concentrations.

Arsenic concentrations varied significantly across the city, with higher levels in the south, central, and northern zones (Kruskal–Wallis test *p* < 0.0001). Median As concentrations in these zones were 10.2, 7.8, and 9.6 ppb, respectively, compared to 2.4 and 0.5 ppb in the east and west (Figure 4). Al concentrations were also significantly different by zone (Kruskal–Wallis test *p* < 0.0001, Figure S2), with higher concentrations in the western zones. In contrast, Pb and Cu concentrations did not exhibit significant geographic differences (Pb Kruskal–Wallis test *p* = 0.11, Figure 4; Cu Kruskal–Wallis test *p* = 0.09, Figure S3).

### 3.3. Effect of Source Water and Provider on Metals Exceedances 

**Figure 5:**
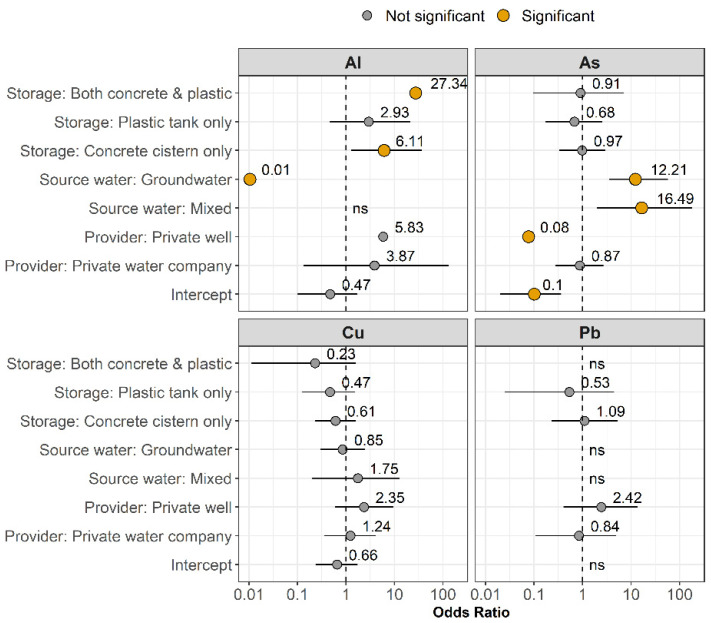
Odds ratios and 95% confidence intervals from multiple logistic regression analyses to evaluate exceedances of the Guatemalan health-based MPL (for Al, As, and Pb) and aesthetic MAL (for Cu). Large orange dots indicate significant predictors. As exceedances were positively associated with groundwater and mixed source waters but negatively associated with private well water. Al exceedances were positively associated with the presence of a concrete cistern but negatively associated with groundwater. Pb and Cu exceedances were not significantly associated with any predictors. Intercept values show the odds of exceedance when all predictors are set to their reference value (see Table S4). Household income, housing type, building age, home ownership, and frequency of water shut offs were not significant in any models. Variables labeled “ns” were not significant and exceeded the plotting boundaries. See Table S4 for more detail.

EMPAGUA customers in the south, central, and northern zones are primarily served by groundwater, while eastern and western zones of the city are also connected to surface water reservoirs. Samples taken from households served by groundwater only were over twelve times as likely to exceed the MPL for As compared to households served by surface water (OR = 12.2, 95% CI: 3.6–57, *p* < 0.001; Figure 5 and Table S4). Households in zones where groundwater and surface water mixed in EMPAGUA’s distribution system were also over 16 times as likely to exceed the MPL for As than surface water alone (OR = 16.5, 95% CI: 2.0–181, *p* = 0.012). Tap water collected from homes supplied by private wells, however, were significantly less likely to exceed the MPL for As compared to municipal sources (OR = 0.08, 95% CI: 0.003–0.47, *p* = 0.02). Samples collected from homes served by private water companies were not more likely to have elevated As compared to municipal waters (OR = 0.87, 95% CI: 0.28–2.67, *p* = 0.81). 

The odds of exceeding the MPL for Al decreased by almost 100% in groundwater compared to surface water (OR = 0.01, 95% CI: 0.0004–0.07, *p* = 0.0001). Furthermore, no significant associations with source water were detected for Pb and Cu. The highest Pb concentration was detected in a private well (42.6 ppb), indicating a high corrosion risk for certain households on well water, but private wells were not associated with elevated Pb overall (OR = 2.42, 95% CI: 0.40–13.4, *p* = 0.31). 

### 3.4. Effect of Water Storage on Metals Exceedances

**Figure 6:**
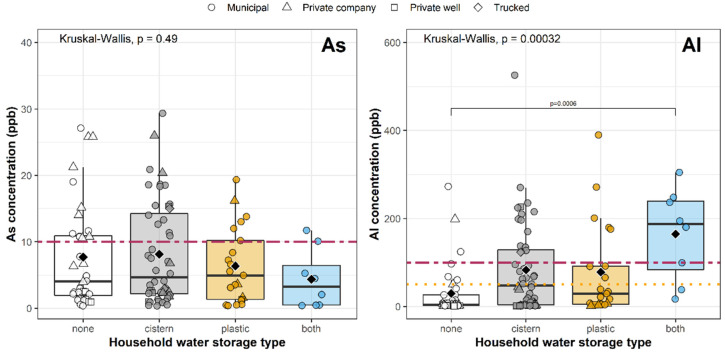
Distribution of As and Al concentrations in first-draw samples from households with and without onsite water storage. Horizontal lines of each box indicate the first quartile, median, and third quartile for each metal; vertical lines indicate the spread of the data up to ± 1.5 times the inter-quartile range. Black diamonds show the mean value. Dashed red lines indicate the MPL for As and Al. Dotted orange line indicates the MAL for Al. Pairwise comparisons highlight significant differences (adjusted for multiple comparisons) between water storage types using Wilcoxon tests. As showed no significant difference between water storage types, but Al did. Kruskal–Wallis tests evaluate the overall significance of water storage on As and Al concentrations.

Most participants (68%) had a concrete cistern or plastic water storage tank connected to their property (Table 1). Local concerns exist that households with onsite water storage could be at greater risk of elevated metals concentrations due to accumulation of sediment in water storage tanks and low frequency of tank cleaning. Notably, the highest As concentration detected (29.3 ppb) was from a household served by municipal groundwater with an underground cistern. However, no significant differences were observed in As concentrations between the various water storage types (Figure 6), nor was the presence of a cistern or plastic tank significantly associated with As exceedances above the MPL after controlling for additional variables (Figure 5). 

The presence of a cistern was positively associated with elevated Al concentrations, however. The odds of exceeding the Al MPL increased by six times among households reporting to have a concrete cistern (OR = 6.1, 95% CI: 1.3–37, *p* = 0.03) and by over 27 times among households with both a cistern and a plastic tank (OR = 27.3, 95% CI: 2.01–946, *p =* 0.03) compared to households with no storage (Figure 5 and Figure 6), possibly due to the highly corrosive effect of concrete on aluminum-bearing components, such as valves or screens, that may be in contact with concrete tanks [44]. The same association was not observed for households with a plastic tank only. 

### 3.5. Occurrence of PFAS in Tap Water 

**Figure 7:**
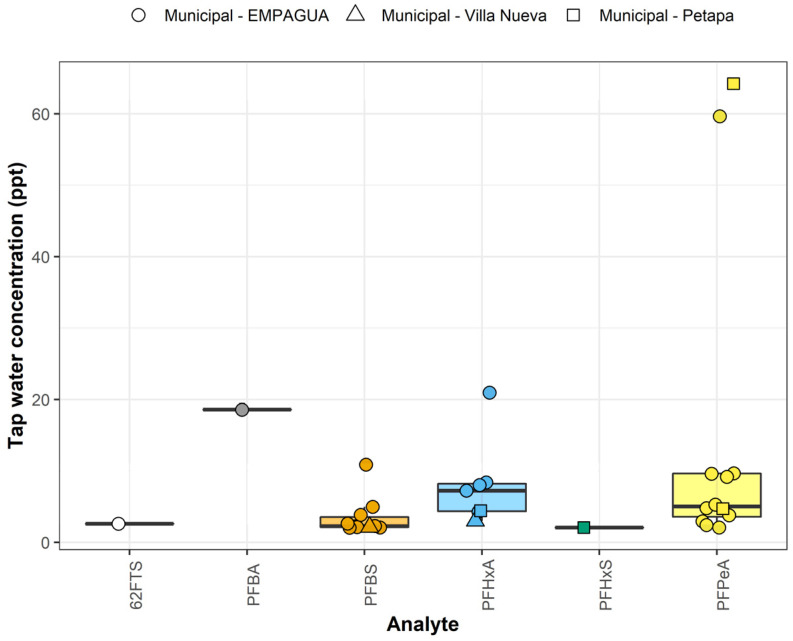
Distribution of individual PFAS analytes detected above the reporting limits in municipal tap waters in the Guatemala City metropolitan area. Horizontal lines of each box indicate the first quartile, median, and third quartile for each metal; vertical lines indicate the spread of the data up to ± 1.5 times the interquartile range from the first and third quartiles, with individual outliers shown.

Low concentrations of six PFAS were detected in some households served by municipal water supplies. No PFAS were detected above the reporting limits in samples from private utilities, private wells, or cisterns served by trucked water. Overall, at least one PFAS was detected above the reporting limit in 19% of samples (*n* = 21). The six PFAS detected, in order of the maximum concentration detected from highest to lowest, were PFPeA, PFHxA, PFBA, PFBS, 6:2 FTS, and PFHxS. Detected concentrations of these six compounds ranged 2.1–64.2 ppt (Figure 7). The sum of all targeted PFAS in the 21 positive samples ranged 2.1–91.5 ppt. No samples exceeded the EPA HAL of 70 ppt for PFOA and PFOS or the EU’s total PFAS limit of 500 ppt, but 6% of samples exceeded the IBWA limit of 10 ppt for the sum of PFAS. PFAS concentrations were not significantly different by zone (Kruskal–Wallis test *p* = 0.13, Figure S4). 

### 3.6. Effect of Water Storage on PFAS

Logistic regression analysis showed no significant effect of source water or service provider on PFAS occurrence. Notably, however, the odds of having any detectable PFAS in the sample was significantly higher among homes reporting the use of a plastic tank on the roof of the home after controlling for water source and service provider (OR_plastic only_ = 6.2, 95% CI: 1.2–48, *p* = 0.04; OR_plastic + cistern_ = 10.6, 95% CI: 1.3–113, *p* = 0.03; Table S5). Concrete cisterns were also positively associated with PFAS, but the odds ratio was not significant (OR = 4.4, 95% CI: 0.99–31, *p* = 0.08). Of the 27 households with plastic tanks, 33% were positive for any targeted PFAS, while only 15% were positive among households without a plastic tank. No other variables, including source water type, provider, frequency of water shutoffs, housing type or age, or household income, were significantly associated with PFAS detections above the reporting limit. 

## 4. Discussion

Rapidly growing metropolitan areas throughout Central America often do not have the resources for advanced water quality testing and treatment. However, chemical monitoring is essential to protect public health, ensure public confidence in water service, and make progress toward Sustainable Development Goal 6 of safe drinking water for all. Thus, this study provides crucial insight into the quality of urban drinking water supplies in Guatemala City, with important implications for metropolitan areas throughout the region.

Out of 113 households, we found that 63% of homes exceeded the Guatemalan health-based MPL for at least one metal, and 40% of homes exceeded the Guatemalan standards for As or Pb alone. Higher As prevalence was associated with municipal groundwater sources, but not with private well water, suggesting an effect of well depth on As concentrations. Groundwater supplies in Guatemala City are drawn from wells ranging 20–600 m deep [11]. Municipal and company-owned wells may be deeper and draw from a different aquifer with more geothermal influence than shallow private wells [45,46]. City zones served by municipal groundwater were more likely to have As concentrations above the MPL. The high prevalence of As above the Guatemalan MPL (33.6%) supports previous findings from Guatemala City [47] and Latin America broadly highlighting geogenic As as a significant health risk [45]. Despite awareness of As risk in the region, there remains a need for enhanced treatment of groundwater (i.e., coagulation and flocculation) for As removal in urban zones [48]. Future research should also evaluate the potential co-benefits of reduced As exposures from expanded use of surface water as a drinking water source, which has been proposed as a strategy to reduce groundwater depletion in the Guatemala City metropolitan area [49]. 

Existing surface water treatment processes also require optimization, however. We found that homes served by surface water were more likely to exceed the Guatemalan health limit for Al, which can be attributed to the standard use of aluminum sulfate as a coagulant during conventional surface water treatment. None of the samples that exceeded the Al MPL also exceeded the As MPL, further indicating that the occurrence of these metals is a function of different source waters and treatment techniques. While monitoring is a first step in understanding urban water quality, water utilities may need governmental support to manage these sources and optimize treatment.

In contrast to As, there was no association between water source or provider and the odds of Pb exceedances at the tap. Elevated Pb concentrations thus appear to be randomly distributed and are likely related to onsite piping and plumbing and/or high corrosivity in certain wells. Very little data exist regarding the presence of Pb service lines in Guatemala City. Although first-draw Pb levels exceeded the Guatemalan MPL in only 8% of households, the high prevalence of detectable Pb (94%) indicates widespread Pb-bearing plumbing components which poses a health risk, particularly for pregnant women, infants, and children [50]. With these findings, water providers and health authorities should consider proactive measures to educate the public regarding the risks of Pb in drinking water and actions that can be taken to reduce water Pb exposures, as well as improve access to Pb-free faucet fixtures and water filters certified to remove Pb at the tap. 

We also detected PFAS in Guatemala City tap water in 19% of household taps. To our knowledge, this is the first study evaluating PFAS in drinking water supplies in Central America. These findings are promising for water providers in Guatemala City in that they indicate relatively good protection of source waters from potential PFAS sources such as industry and wastewater, despite estimates that 95% of the country’s wastewater goes untreated [10]. Interestingly, however, the occurrence of PFAS was significantly associated with the use of plastic water storage tanks. High-density polyethylene plastic products treated with fluorine for stability [51] and/or recycled plastic containers [52] could leach PFAS into stored water. Overall, 68% of participants had some form of water storage onsite. Further study is needed to understand the potential chemical risks of domestic water storage in concrete cisterns and plastic tanks, including PFAS and metals, which are widely used to minimize interruptions in water service worldwide [53]. Future research could also evaluate onsite water storage systems for additional chemical risks, such as asbestos cement [54]. 

A key limitation of this study was that recruitment on a university campus led to the selection of households with higher education levels and socioeconomic status. Tap water in these households may not be representative of the city overall; different or additional factors may be associated with water quality in lower-income neighborhoods that may have different housing characteristics and/or be supplied by informal water providers. Importantly, however, this study demonstrates that participatory science can be an efficient way to conduct water quality sampling in burgeoning urban zones to provide individuals, utilities, and public health authorities with important water quality information. With mounting pressure on FEW resources, policies and financial resources that support sustainable and health-protective water service in Guatemala City will become increasingly important. 

## 5. Conclusions

In this study, tap water samples were collected at 113 households in the Guatemala City metropolitan area using a participatory science approach. Water quality results were correlated to socioeconomic, infrastructural, and geospatial information to evaluate risk factors associated with the presence of chemical contaminants. Results showed a high prevalence of As, Al, and Pb in tap water across the city, and indicated a range of sources of contamination, including geogenic, water treatment-related, and premise plumbing sources. Household water storage practices, frequently used to manage intermittent water service, may also introduce additional contaminants such as PFAS and Al into household tap water. The results provide actionable information for individuals, public health authorities and water service providers seeking to reduce drinking water-related exposures. As climate change and urbanization present increasing environmental health challenges in Latin America, engaging communities as participants in the scientific process can help generate evidence that supports healthy and sustainable development throughout the region. 

## Figures and Tables

**Table 1:** Water service and household characteristics of the study population (total *n* = 113).

	*n*	%
**Water service provider**		
Municipal—EMPAGUA	70	62%
Municipal—Villa Nueva	5	4%
Municipal—San Miguel Petapa	4	4%
Private water company	20	18%
Private well	12	11%
Trucked water ^a^	2	2%
**Water source type**		
Surface water	36	32%
Ground water	70	62%
Mixed	5	4%
Unknown	2	2%
**Frequency of water shut offs**		
Once a year or less	44	39%
A few times a year	32	28%
A few times a month	8	7%
A few times a week	15	13%
Daily	14	12%
**Housing type**		
Apartment	11	10%
Formal house	100	88%
Improvised house	1	1%
Room	1	1%
**Water storage**		
Concrete cistern only	48	42%
Plastic tank only	21	19%
Both cistern + plastic	8	7%
None	36	32%
**Home ownership**		
Own	92	81%
Rent	21	19%
**Age of home (years)**		
≤10	10	9%
11–20	17	15%
21–30	25	22%
31–40	9	8%
>40	29	26%
Not reported or unknown	23	20%
**Monthly household income (Q)**		
≤4000	5	4%
4001–8000	17	15%
8001–16,000	26	23%
16,001–24,000	19	17%
>24,000	34	30%
Not reported	12	11%
**Level of education attained**		
No schooling	2	2%
Elementary school	1	1%
Middle school	1	1%
High school	12	11%
University or above	97	86%

^a^—Private truckers may serve individual households directly or deliver to a community storage tank in areas that municipal lines do not yet reach [41]. The source of trucked water was unknown.

## Data Availability

The data presented in this study may be made available on request from the corresponding author. The data are not publicly available due to participant confidentiality.

## Supplementary Materials

The following supporting information can be downloaded at: https://www.mdpi.com/article/10.3390/ijerph19106004/s1, Table S1: Metals analyzed under U.S. EPA method 200.8; Table S2: PFAS analyzed under U.S. EPA method 533; Table S3: Summary of metals concentrations in Guatemala City first draw tap water samples across all water service providers and source water types; Table S4: Multiple logistic regression results to evaluate variables associated with any detections of Al, Al, Pb, and Cu above MPLs (Al, As, Pb) and MAL (Cu) in tap water samples; Table S5: Multiple logistic regression results to evaluate variables associated with any PFAS detections in tap water samples; Figure S1: A copy of the study flyers in English and Spanish, which were available online, emailed to eligible participants with UVG email addresses, and also printed for the UVG maintenance staff; Figure S2: Distribution of Al concentrations in first-draw tap water throughout the Guatemala City metropolitan area; Figure S3: Distribution of Cu concentrations in first-draw tap water throughout the Guatemala City metropolitan area; Figure S4: Distribution of the sum of PFAS detected throughout the Guatemala City metropolitan area.
