# Supplementary material for: A Participatory Science Approach to Evaluating Factors Associated with the Occurrence of Metals and PFAS in Guatemala City Tap Water
Source: Int J Environ Res Public Health. 2022 May 15;19(10):6004. doi: 10.3390/ijerph19106004 (PMC9142002; doi:10.3390/ijerph19106004)

Supplementary Information

A Participatory Science Approach to Evaluating Factors Associated with the Occurrence of Metals and PFAS in Guatemala City Tap Water

Jennifer Hoponick Redmon[1*], Riley Mulhern[1*], Edwin Castellanos[2], Erica Wood[1], Andrea McWilliams[1], Isabel Herrera[2], Chamindu Liyanapatirina[1], Frank Weber[1], Keith Levine[1], Evan Thorp[1], Nichole Bynum[1], Kelly Amato[1], Maria Andrea Najera Acevedo[2], Justin Baker[1], George Van Houtven[1], Candise Henry[1], Christopher Wade[1], AJ Kondash[1]

1 - RTI International, Research Triangle Park, North Carolina, United States of America

2 - Observatorio Económico Sostenible, Universidad del Valle de Guatemala, Guatemala.

*Corresponding Authors. These authors contributed equally to this work.
Jennifer Hoponick Redmon (jredmon@rti.org) and Riley Mulhern (rmulhern@rti.org)

Additional Study Methods Information

Recruitment and selection

Our study design included the use of participatory science—the collection of water samples by faculty, staff, and students affiliated with Universidad del Valle de Guatemala (UVG)—along with the subsequent analysis of data as part of a collaborative project with professional scientists at RTI International and professors and graduate researchers at UVG. The study flyer was available online and was emailed to those affiliated with UVG (students and staff), and shown on campus in select places (e.g., maintenance staff area). Interested participants could click on the link to fill out a preliminary interest survey, while UVG maintenance staff completed their interest questionnaire with a UVG researcher while following COVID-19 safety protocols. The English and Spanish versions are shown below as Figure S1.

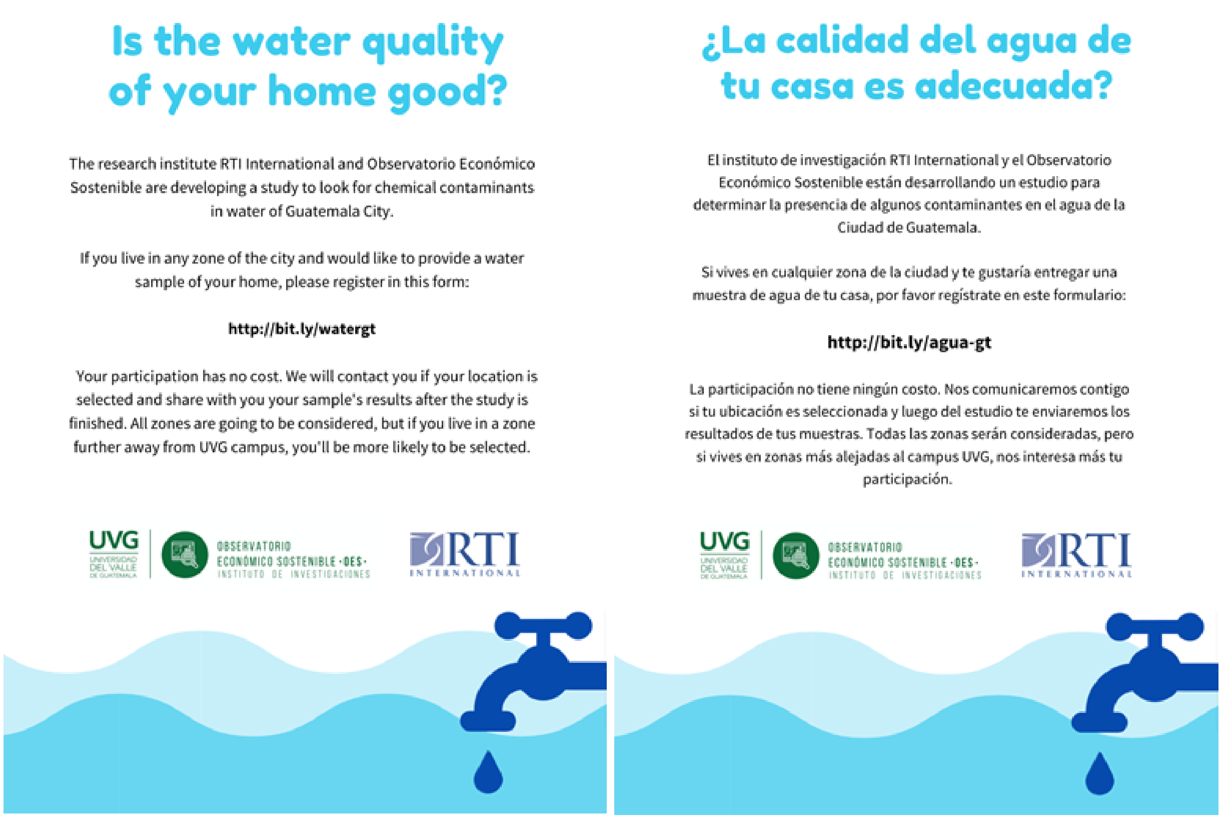

Figure S1. A copy of the study flyers in English and Spanish, which were available online, emailed to eligible participants with UVG email addresses, and also printed for the UVG maintenance staff.

Interested participants answered preliminary eligibility questions either verbally or through an online form regarding their home's location in the city along with their ability to participate in the sampling process. This community-engaged approach was critical to the success of the study given restrictions associated with the COVID-19 pandemic. We also adapted to COVID-19 restrictions by conducting virtual meetings, along with online enrollment and training.

While many potential participants voiced interest using our online survey, we also conducted on-site, COVID-safe support for interested maintenance and janitorial staff with limited internet access to be

inclusive of variation in geographical areas, socioeconomic status, and technological access and capability. A total of 207 potential participants were identified. All participants living in zones/municipalities with seven or fewer potential participants in that zone were included. In the zones/municipalities with more than seven potential participants (zones 2, 10, 11, 15, 16, and Villa Nueva), we selected seven participants per zone with final participants chosen if they met the final selection criteria (i.e., no plan to change water source in next few months, currently can travel to UVG campus, then can pick up and return test kit to UVG). We retained all potential participants in zone 18 (n=12) and Villa Nueva (n=13) to account for the larger population in those areas according to census data. In total, we enrolled 120 participants using this tiered approach representing 20 of the 22 formal city zones and the municipalities of Villa Nueva, San Miguel Petapa, and Villa Canales. Selected participants were then provided with instructions for arranging pickup (or delivery by courier) of their sampling kit.

Training

After the kits were delivered by courier or picked up on UVG's campus (based on the participant's preference), participants received an email or text message with a reminder to take their samples, written instructions, and a link to an instructional video at https://www.youtube.com/watch?v=a_KsN6W77U8. Each kit included detailed paper instructions on how to collect the water samples, chain of custody documentation, a pair of nitrile gloves, one 250 mL HDPE sampling bottle for metals, one 250 mL polypropylene sampling bottle with 250 mg of ammonium acetate as a preservative for PFAS, and two additional 250 mL polypropylene sampling bottles (one empty with preservative, one with reagent water) for collecting a PFAS field blank. Participants were instructed to select a tap without a point-of-use or under-sink filter installed. Participants could also contact research staff by phone or email for additional support if there were any questions or comments.

Sampling procedure

First, participants collected a first-draw sample after at least 8 hours of stagnation time in the HDPE bottle, typically the morning after receiving the sampling kit. Next, the water was run for 3-5 minutes (if participants reported not having enough water to run water for this long continuously, they were instructed to omit this step) and the first 250 mL polypropylene bottle, containing ammonium acetate powder as a preservative per EPA 533, was filled. Finally, participants poured the field blank reagent water into the remaining empty sampling bottle as a field blank. The three filled sample bottles were then stored in a refrigerator until being delivered to UVG to our study research coordinator, or until a courier picked them up. Out of the 120 selected participants, five did not pick up their test kit (4%) and two kits were not returned (1.6%), resulting in 113 total households sampled. Samples were stored under refrigeration at UVG while waiting to be shipped. Sample kits were placed into FedEx cooling boxes by our study research coordinator and shipped back to RTI International in Research Triangle Park, NC, US.

Contaminants Considered in Study

Metals

The study included analysis of the following metals, as shown in Table S1. Metals such as arsenic and chromium can be naturally occurring in water sources. Metals such as lead and copper are typically present in tap water from piping and plumbing. Aluminum can be present as a byproduct of water treatment. Metals can also be indicative of industrial pollution in certain cases.

Table S1. Metals analyzed under U.S. EPA method 200.8.

Analyte	Abbreviation	CAS#
Aluminum	Al	7429-90-5
Antimony	Sb	7440-36-0
Arsenic	As	7440-38-2
Barium	Ba	7440-39-3
Beryllium	Be	7440-41-7
Cadmium	Cd	7440-43-9
Chromium	Cr	7440-47-3
Cobalt	Co	7440-48-4
Copper	Cu	7440-50-8
Lead	Pb	7439-92-1
Manganese	Mn	7439-96-5
Mercury	Hg	7439-97-6
Nickel	Ni	7440-02-0
Selenium	Se	7782-49-2
Silver	Ag	7440-22-4
Thallium	Tl	7440-28-0
Thorium	Th	7440-29-1
Uranium	U	7440-61-1
Vanadium	V	7440-62-2
Zinc	Zn	7440-66-6

PFAS

PFAS are synthetic chemicals used to make nonstick, water and oil resistant consumer products and firefighting foam. They are nicknamed “Forever Chemicals” because of persistence in the environment and bioaccumulation in living organisms. They are potentially toxic at low levels and may be in tap water from industrial, agricultural, and residential use of PFAS-containing products.

Table S2. PFAS analyzed under U.S. EPA method 533.

Analyte	Chemical formula	Chain length	CAS#	Chemical name
**Perfluorosulfonic acids (PFSAs)**				
PFBS	C4HF9O3S	4	375-73-5	Perfluorobutane sulfonic acid
PFPeS	C5HF11O3S	5	2706-91-4	Perfluoropentane sulfonic acid
PFHxS	C6HF13O3S	6	355-46-4	Perfluorohexane sulfonic acid
PFHpS	C7HF15O3S	7	375-92-8	Perfluoroheptane sulfonic acid
PFOS	C8HF17O3S	8	1763-23-1	Perfluorooctane sulfonic acid
**Perfluorocarboxylic acids (PFCAs)**				
PFBA	C4HF7O2	4	375-22-4	Perfluorobutanoic acid
PFPeA	C5HF9O2	5	2706-90-3	Perfluoropentanoic acid
PFHxA	C6HF11O2	6	307-24-4	Perfluorohexanoic acid
PFHpA	C7HF13O2	7	375-85-9	Perfluoroheptanoic acid
PFOA	C8HF15O2	8	335-67-1	Perfluorooctanoic acid
PFNA	C9HF17O2	9	375-95-1	Perfluorononanoic acid
PFDA	C10HF19O2	10	335-76-2	Perfluorodecanoic acid
PFUnA	C11HF21O2	11	2058-94-8	Perfluoroundecanoic acid
PFDoA	C12HF23O2	12	307-55-1	Perfluorododecanoic acid
**Perfluoroalkyl ether acids (PFEAs)**				
PFMPA	C4HF7O3	4	377-73-1	Perfluoro-3-methoxypropanoic acid
PFEESA	C4HF9O4S	4	113507-82-7	Perfluoro(2-ethoxyethane)sulfonic acid
NFDHA	C5HF9O4	5	151772-58-6	Nonafluoro-3,6-dioxaheptanoic acid
PFMBA	C5HF9O3	5	863090-89-5	Perfluoro-4-methoxybutanoic acid
HFPO-DA	C6HF11O3	6	13252-13-6	Hexafluoropropylene oxide-dimer acid
ADONA	C7H2F12O4	7	919005-14-4	4,8-Dioxa-3H-perfluorononanoic acid
9ClPF3ONS	C8HClF16O4S	8	756426-58-1	9-Chlorohexadecafluoro-3-oxanonane-1-sulfonic acid
11ClPF3OUdS	C10HClF20O4S	10	763051-92-9	11-Chloroeicosafluoro-3-oxaundecane-1-sulfonic acid
**Fluorotelomer sulfonates**				
4:2 FTS	C6H5F9O3S	6	757124-72-4	1H,1H, 2H, 2H-Perfluorohexane sulfonic acid
6:2 FTS	C8H5F13O3S	8	27619-97-2	1H,1H, 2H, 2H-Perfluorooctane sulfonic acid
8:2 FTS	C10H5F17O3S	10	39108-34-4	1H,1H, 2H, 2H-Perfluorodecane sulfonic acid

Additional Results Information

Table S3. Summary of metals concentrations in Guatemala City first draw tap water samples across all water service providers and source water types. Concentrations shown in parts per billion (ppb).

Element	Mean	Max	Min	Median	90th perc.	MPL	MAL	*n* above MLP	% above MLP	*n* above MLA	% above MLA	% above reporting limit
**Al**	**70.8**	**525.7**	**1.0**	**21.3**	**214**	**100**	**50**	**27**	**24%**	**45**	**40%**	**100%**
**As**	**7.4**	**29.3**	**0.3**	**4.1**	**18.6**	**10**	**-**	**38**	**34%**	**-**	**-**	**100%**
Ba	59.7	159.1	2.6	49.4	104	700	-	0	0%	-	-	100%
Be	0.1	0.1	0.1	0.1	0.07	-	-	-	-	-	-	0%
Cd	0.1	1.8	<0.1	0.0	0.13	3	-	0	0%	-	-	13%
Co	0.2	1.7	<0.1	0.1	0.61	-	-	-	-	-	-	45%
Cr	0.3	2.4	<0.5	0.2	0.61	50	-	0	0%	-	-	19%
Cu	56.8	947.4	0.3	24.8	120	1500	50	0	0%	35	31%	100%
Fe	110.6	4849.8	<1	19.3	220	-	300	-	-	5	4%	96%
Hg	0.1	0.2	<0.1	0.1	0.10	1	-	0	0%	-	-	13%
Mn	10.7	398.7	<0.1	2.9	20.2	400	100	0	0%	2	2%	98%
**Pb**	**2.9**	**42.6**	**<0.1**	**0.7**	**7.5**	**10**	**-**	**10**	**9%**	**-**	**-**	**94%**
Sb	0.2	0.6	<0.1	0.2	0.40	-	-	-	-	-	-	77%
Se	0.7	1.2	<1	0.7	0.71	10	-	0	0%	-	-	1%
Sn	0.9	66.4	<0.5	0.0	0.41	-	-	-	-	-	-	9%
Th	0.1	0.4	<0.2	0.1	0.11	-	-	-	-	-	-	4%
Tl	0.0	0.3	<0.1	0.0	0.09	-	-	-	-	-	-	6%
U	0.3	1.9	<0.1	0.2	0.60	100	-	0	0%	-	-	67%
V	9.2	23.4	<5	8.5	15.1	-	-	-	-	-	-	51%
Zn	398.7	3608.1	4.0	152.6	1016	70000	3000	0	0%	1	1%	67%

Table S4. Multiple logistic regression results to evaluate variables associated with any detections of Al, Al, Pb, and Cu above MPLs (Al, As, Pb) and MAL (Cu) in tap water samples. Significant predictors shown in blue. OR=Odds Ratio.

		**Al**		**As**		**Cu**		**Pb**	
		**OR**	**95% CI**	**OR**	**95% CI**	**OR**	**95% CI**	**OR**	**95% CI**
	Intercept	0.47	0.10–1.7	**0.10**	**0.02–0.36**	0.66	0.24–1.8	0.0	NA*
**Water provider**	Municipal provider	*Reference*							
	Private company	3.87	0.13–132	0.87	0.28–2.67	1.24	0.36–4.1	0.8	0.11–4.9
	Private well	5.83	0.20–204	**0.08**	**0–0.47**	2.35	0.59–9.5	2.4	0.40–13.4
**Source water**	Surface water	*Reference*							
	Mixed	0.00	NA*	**16.5**	**2.0–181**	1.75	0.2–13	6.1 x10^7	NA*
	Groundwater	**0.01**	**0–0.07**	**12.2**	**3.6–57**	0.85	0.29–2.5	3.3x10^7	NA*
**Storage**	No storage	*Reference*							
	Both	**27.3**	**2.01–946**	0.91	0.1–7.0	0.23	0.01–1.6	0.0	NA*
	Cistern	**6.11**	**1.3–37**	0.97	0.33–2.9	0.61	0.23–1.6	1.1	0.23–5.3
	Plastic tank	2.93	0.47–21	0.68	0.17–2.5	0.47	0.13–1.6	0.5	0.02–4.5

**Exact confidence intervals could not be calculated for these regression coefficients due to too few observations above the MPL for the category of the predictor variable.*

Table S5. Multiple logistic regression results to evaluate variables associated with any PFAS detections in tap water samples. Significant predictors shown in blue. OR=Odds Ratio.

**Independent variable**	**OR**	**95% Confidence Interval**	**P-value**
**Intercept**	**0.09**	**0.02-0.34**	**0.002**
**Water provider**			
Municipal provider	*reference*		
Private water company	0.89	0.17–4.0	0.89
Private well	4.3x10^{-8}	0–2x10^{38}	0.99
**Source type**			
Surface water	*reference*		
Mixed source water	2.1	0.2–16	0.48
Groundwater	1.1	0.3–3.8	0.84
**Water storage**			
No storage	*reference*		
Cistern	4.4	0.99–31	0.08
**Plastic tank**	**6.2**	**1.2–48**	**0.02**
**Both**	**10.6**	**1.3–113**	**0.03**

Figure S2. Distribution of Al concentrations in first-draw tap water throughout the Guatemala City metropolitan area. Horizontal lines of each box indicate the first quartile, median, and third quartile for each metal; vertical lines indicate the spread of the data up to ± 1.5 times the interquartile range from the first and third quartiles, with individual outliers shown. Dashed red line indicates the MPL for Al; dotted orange line indicates the MAL for Al. Pairwise comparisons highlight significant differences (adjusted for multiple comparisons) between geographic areas using Wilcoxon tests. Kruskal-Wallis test evaluates the overall significance of the geographic area on Al concentrations.

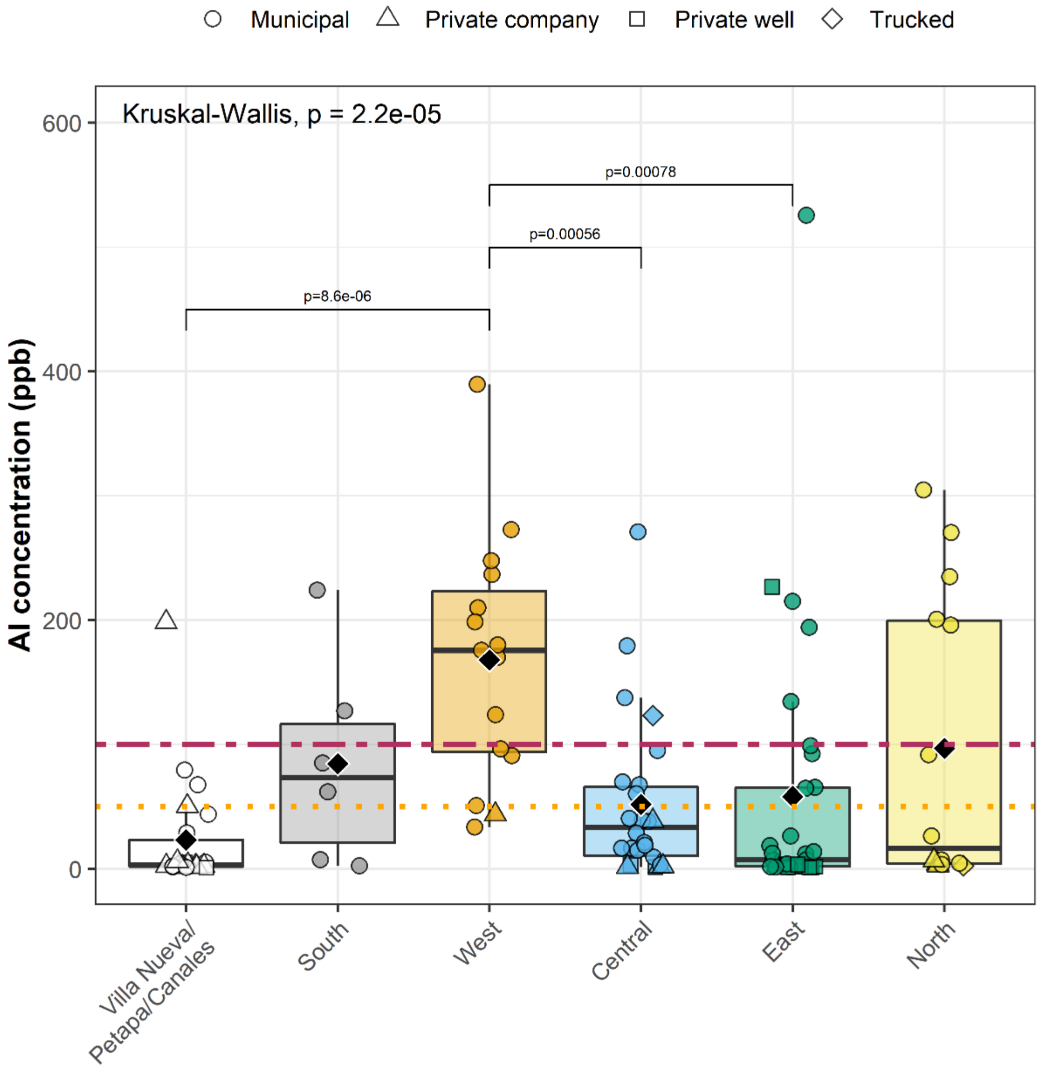

Figure S3. Distribution of Cu concentrations in first-draw tap water throughout the Guatemala City metropolitan area. Horizontal lines of each box indicate the first quartile, median, and third quartile for each metal; vertical lines indicate the spread of the data up to ± 1.5 times the interquartile range from the first and third quartiles, with individual outliers shown. Dashed red line indicates the MPL for Cu; dotted orange line indicates the MAL for Cu. Kruskal-Wallis test evaluates the overall significance of the geographic area on Cu concentrations.

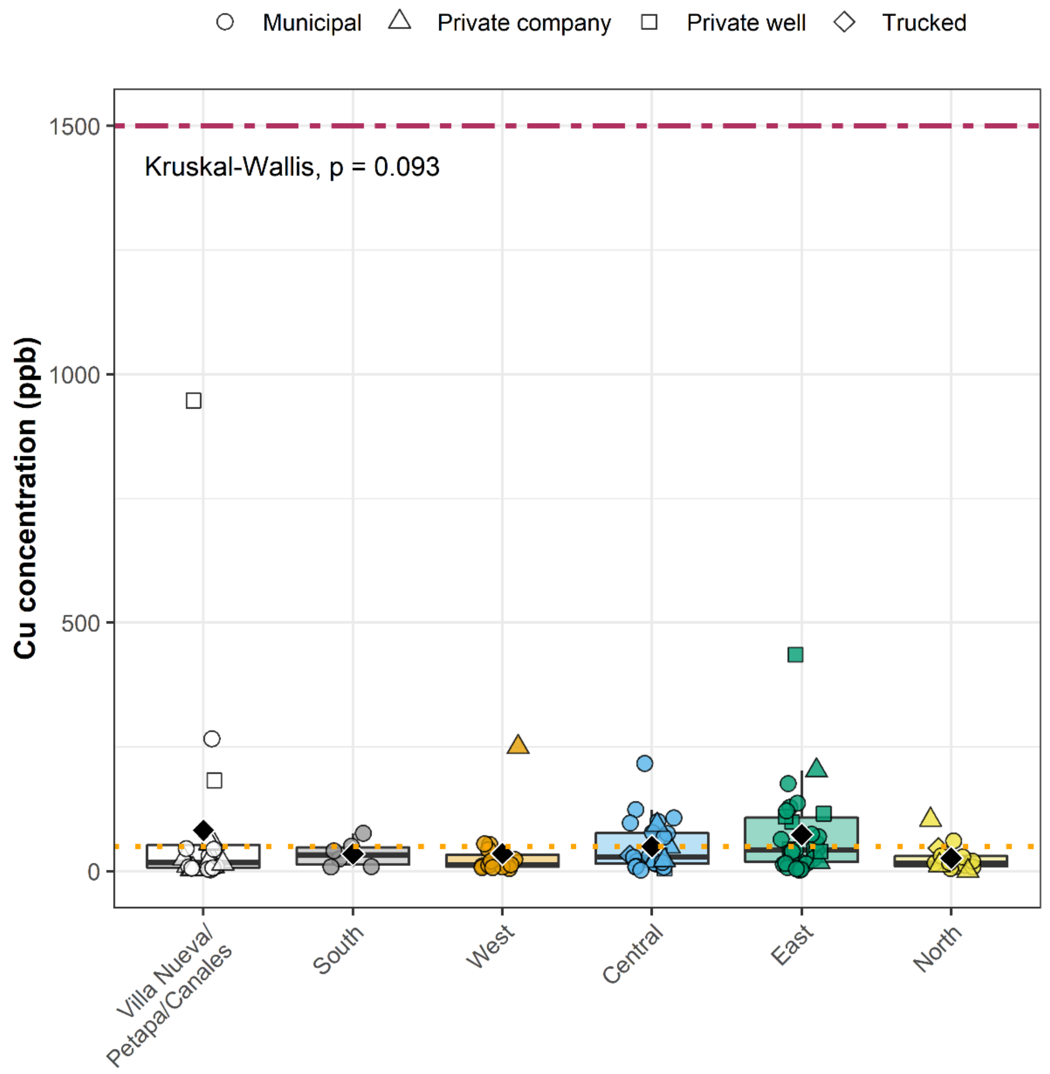

Figure S4. Distribution of the sum of PFAS detected throughout the Guatemala City metropolitan area. Samples below reporting limits not shown. Horizontal lines of each box indicate the first quartile, median, and third quartile for each metal; vertical lines indicate the spread of the data up to ± 1.5 times the interquartile range from the first and third quartiles, with individual outliers shown. Kruskal-Wallis test evaluates the overall significance of the geographic area on PFAS concentrations.

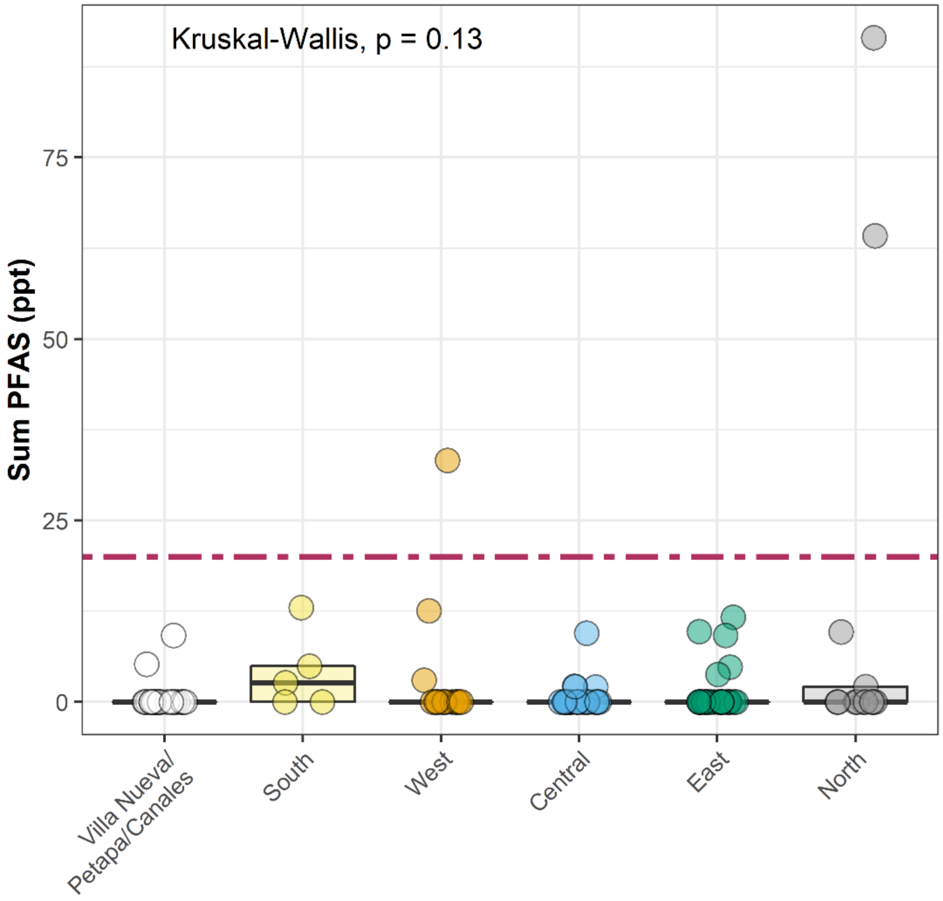

Supplement: Supplementary file 1 [file ijerph-19-06004-s001.zip › ijerph-1710600-supplementary.pdf]
